# Host range expansion of *Leucobacter holotrichiae*: first evidence of mammalian infection and comparative genomic insights

**DOI:** 10.3389/fmicb.2025.1716137

**Published:** 2025-12-11

**Authors:** Yong Jian Li, Bo Rui Qi, Shu Zhu Cao, Run Ze Zhang, Long Ling Jiao, Ming Zhou, Jin Chun Cai, Meng Ying Du, Ke Shuang Li, Chen Cheng Xiao, Ya Yin Qi

**Affiliations:** College of Animal Science and Technology, Shihezi University, Shihezi, Xinjiang, China

**Keywords:** *Leucobacter holotrichiae*, isolation and identification, microbiological characterization, antimicrobial susceptibility, biofilm formation, murine infection model, bovine actinomycosis, whole-genome sequencing

## Abstract

*Leucobacter holotrichiae* has previously been identified exclusively in insect hosts, with no reports of its presence in mammals. This study is the first to report the isolation of three *L. holotrichiae* strains (LH23001, LH23002, and LH23003) from mixed cultures of bovine actinomycotic abscesses in a large-scale dairy farm in Xinjiang, China. Phenotypic analysis revealed that these Gram-positive bacilli are non-spore-forming, non-flagellated, non-hemolytic, non-motile, and capable of biofilm formation. The isolates formed transparent membranous white colonies on modified Gao’s medium and could grow in BHI liquid medium containing 9% NaCl. Phylogenetic analysis based on 16S rRNA gene sequences and average nucleotide identity (ANI) further confirmed that these strains are most closely related to *L. holotrichiae*, with all strains exhibiting strong biofilm-forming ability. Intraperitoneal infection experiments in mice showed that infection induced pathological changes in multiple tissues: vacuolar degeneration of cardiomyocytes, mild steatosis of hepatocytes, focal necrosis of a small number of lymphocytes in the white pulp of the spleen, extensive granulocyte infiltration in the alveolar walls of lung tissue, and mild edema of renal tubular epithelial cells in the renal cortex. Whole-genome sequencing results indicated that the genome size of these strains ranges from 3.63 to 3.68 Mb with a GC content of 66.8–67.2%. They carry multiple antimicrobial resistance genes and virulence factors, and five complete prophages were predicted. Functional annotation results showed that the strains have annotated information in databases including NR, GO, eggNOG, Swiss-Prot, CAZy, CARD, and VFDB. This study expands the known host range of *L. holotrichiae*, systematically analyzes its biological characteristics and genomic features, and provides a theoretical basis for future research on this bacterium.

## Introduction

The genus *Leucobacter*, a member of the family *Microbacteriaceae* within the phylum Actinobacteria (Takeuchi et al., 1996), comprises Gram-positive, non-spore-forming bacteria with high GC content. Species within this genus are typically isolated from diverse environmental habitats (Sturm et al., 2011), including soil (Zhu et al., 2022), fermented seafood (Shin et al., 2011), Skin (Boxberger et al., 2023), plant rhizospheres (Li et al., 2024), and the intestinal tracts of insects (Hyun et al., 2021), and are generally regarded as non-pathogenic or commensal organisms. Since its initial description from the gut of *Holotrichia oblita* larvae, *Leucobacter holotrichiae* has been exclusively associated with invertebrate hosts (Zhu et al., 2016). To date, no reports have documented its presence in vertebrates or its involvement in mammalian disease.

In recent years, the ecological and pathogenic boundaries of environmental actinobacteria have become increasingly ambiguous (Poullain and Nuismer, 2012; Barber and Fitzgerald, 2024). Members of genera once considered strictly saprophytic are now being detected in clinical and veterinary settings. This shift is driven in part by global changes in microbiome composition, antimicrobial usage, host immune status, and environmental pressures—factors that collectively contribute to the emergence of cryptic or opportunistic pathogens (Berendonk et al., 2015). In this context, this study is the first to successfully isolate this bacterium from bovine actinomycotic abscesses, marking its ecological niche expansion from insects to mammals. This finding not only confirms the evolutionary trend of pathogenic potential in environmental actinomycetes but also highlights the need to re-evaluate the host adaptation range and pathogenic mechanisms of such microorganisms (Wani et al., 2022; Combs et al., 2023; Duar et al., 2017).

In veterinary medicine, actinomycosis is a chronic suppurative infection characterized by firm abscesses and granulomatous inflammation (Brook, 2008; Figueiredo et al., 2013). While *Actinomyces bovis* has traditionally been considered the primary causative agent in cattle, emerging evidence underscores the contribution of polymicrobial communities and secondary colonizers to disease persistence and progression (Duar et al., 2017). Bovine abscesses have been documented in several cases as polymicrobial infections; however, the isolation of *L. holotrichiae* from bovine actinomycotic lesions reported here is the first of its kind. This study focuses specifically on the isolation and identification of *L*. *holotrichiae* and aims to elucidate its potential role in such mixed infections. The isolation of *L*. *holotrichiae* from actinomycotic lesions in dairy cattle thus represents a notable intersection of environmental microbiology and veterinary infectious disease, meriting further in-depth investigation.

In this study, we report the isolation and comprehensive characterization of three *L. holotrichiae* strains from actinomycotic abscesses in dairy cattle in Xinjiang, China. To assess their biological behavior and potential for mammalian infection, we conducted an integrated series of phenotypic, microbiological, and genomic analyses. These included biochemical profiling, antimicrobial susceptibility testing, biofilm formation assays, experimental infection in a murine model, and whole-genome sequencing with comparative annotation. Through this multifaceted approach, we aimed to (i) validate the taxonomic identity of the isolates, (ii) evaluate their capacity to colonize and disseminate within a mammalian host, and (iii) elucidate the genomic features that may facilitate their apparent host range expansion. The results demonstrate that *L. holotrichiae*, as a potential opportunistic pathogen, resides in the suppurative lesions of mammals (cattle), significantly expanding its known host range from insects to livestock. This study systematically analyzed the bacterium’s biological phenotypes, pathogenic potential, and genomic basis, revealing key pathogenicity-related traits including environmental tolerance, biofilm formation, and partial resistance to antibiotics. This finding provides new empirical evidence for the notion that the ecological and pathogenic boundaries of environmental actinobacteria are increasingly blurred.

## Materials and methods

### Bacterial isolation and identification

Purulent samples were aseptically collected from mandibular actinomycotic abscesses of three adult Holstein dairy cows with chronic swelling and suppuration in Xinjiang, China, during routine veterinary inspections in 2023. Each sample was streaked onto brain heart infusion (BHI) agar plates and incubated aerobically at 37 °C for 24–48 h. Colonies with similar morphology—dry, pale-yellow, and slightly rough—were selected and sub-cultured to purity. For molecular identification, genomic DNA was extracted from pure cultures using a commercial bacterial genomic DNA extraction kit (Tiangen, China), following the manufacturer’s protocol. The nearly full-length 16S rRNA gene was amplified using universal primers 27F (5’-AGAGTTTGATCCTGGCTCAG-3′) and 1492R (5’-GGTTACC TTGTTACGACTT-3′) (Greisen et al., 1994). PCR was carried out in a 25 μL reaction volume with an initial denaturation at 94 °C for 5 min, followed by 30 cycles of 94 °C for 30 s, 55 °C for 30 s, and 72 °C for 90 s, and a final extension at 72 °C for 7 min. Amplicons were verified by 1% agarose gel electrophoresis and submitted for bidirectional Sanger sequencing (Sangon Biotech, China). The obtained sequences were analyzed using the NCBI BLASTn tool to identify closely related taxa. Phylogenetic trees were constructed using the neighbor-joining method with 1,000 bootstrap replications in MEGA 11 software. All three isolates (designated LH23001, LH23002, and LH23003) exhibited 99.7–99.9% identity with *Leucobacter holotrichiae* (KJ_461711.1) (Zhu et al., 2016), confirming their taxonomic affiliation.

### Morphological, biochemical, and physiological characterization

Colonial morphology was assessed after aerobic incubation of isolates on BHI agar plates at 37 °C for 24 h. Colony color, texture, elevation, and margin were recorded. Cellular morphology was observed using Gram staining under a light microscope (1,000× magnification). All isolates were Gram-positive, rod-shaped, and non-spore-forming. Motility was evaluated using a semi-solid motility medium (0.4% agar supplemented with 5% fetal bovine serum). Each isolate was stab-inoculated and incubated at 37 °C for 24–48 h. The absence of radial diffusion from the inoculation site indicated non-motility. Catalase activity was determined by adding 3% hydrogen peroxide to freshly cultured bacterial cells on a glass slide. The appearance of rapid bubble formation was interpreted as a positive reaction. Oxidase activity was tested using oxidase reagent strips (bioMérieux, France), with color change to deep purple within 30 s indicating a positive result. Carbohydrate utilization profiles were evaluated using the API 50 CH system (bioMérieux, France). Bacterial suspensions were prepared in API 50 CHL medium to a turbidity equivalent of McFarland 2.0, and inoculated into microtubes. Strips were incubated at 37 °C for 48 h and color changes were used to assess fermentation patterns according to the manufacturer’s instructions. Enzymatic activity was assessed with the API ZYM system (bioMérieux, France). Suspensions of each isolate were inoculated into wells containing chromogenic enzyme substrates. Reactions were monitored at 4 h and 24 h intervals. All biochemical and enzymatic tests were conducted in triplicate for validation and reproducibility.

### Antibiotic susceptibility testing

Antimicrobial susceptibility of the three *L. holotrichiae* isolates (LH23001, LH23002, and LH23003) was evaluated using the standard disk diffusion method (Kirby–Bauer) on Mueller-Hinton agar (Oxoid, United Kingdom), following the Clinical and Laboratory Standards Institute (CLSI) guidelines (M100, 2023). Overnight cultures were adjusted to a turbidity equivalent to 0.5 McFarland standard and uniformly spread onto agar plates using sterile swabs. Commercial antibiotic disks (Hangwei, China) were placed onto the inoculated plates, including the following agents: ampicillin (10 μg), amoxicillin–clavulanate (20/10 μg), ceftiofur (30 μg), streptomycin (10 μg), tetracycline (30 μg), gentamicin (10 μg), florfenicol (30 μg), ciprofloxacin (5 μg), enrofloxacin (5 μg), sulfamethoxazole-trimethoprim (23.75/1.25 μg), and erythromycin (15 μg). Plates were incubated at 37 °C for 18–24 h, and the diameters of inhibition zones were measured in millimeters. The interpretation of the results was mainly based on the criteria for aerobic actinomycetes in CLSI document M24. For antibiotics not covered in M24, we refer to the general recommendations for rare bacteria in the CLSI document M45 and determine the results as Susceptible (S), moderately Susceptible (I) or resistant (R) (Abbey and Deak, 2019). All susceptibility tests were performed in triplicate to ensure consistency.

### Biofilm formation assay

The ability of *L. holotrichiae* isolates to form biofilms was assessed using the crystal violet microtiter plate assay. Overnight cultures of LH23001, LH23002, and LH23003 were diluted 1:100 in fresh brain heart infusion (BHI) broth and 200 μL of each suspension was inoculated into sterile, flat-bottom 96-well polystyrene microplates (Corning, United States). Each strain was tested in eight technical replicates. *Staphylococcus aureus* ATCC 6538 and uninoculated BHI medium were used as positive and negative controls, respectively. The plates were incubated aerobically at 37 °C for 24 h without agitation. After incubation, wells were gently washed three times with sterile phosphate-buffered saline (PBS, pH 7.4) to remove planktonic cells. Attached biofilms were fixed with 200 μL of 99% methanol for 15 min, air-dried, and stained with 0.1% crystal violet solution for 15 min. Excess dye was rinsed off with distilled water, and the retained stain was solubilized with 200 μL of 33% glacial acetic acid. The optical density (OD) was measured at 595 nm using a microplate reader (BioTek, United States). Based on OD values, biofilm-forming capacity was classified as follows: OD ≤ ODc, no biofilm (−); ODc < OD ≤ 2 × ODc, weak (+); 2 × ODc < OD ≤ 4 × ODc, moderate (++); OD > 4 × ODc, strong (+++), where ODc represents the cut-off value calculated as the mean OD of the negative control plus three standard deviations. Each assay was repeated three times independently to ensure reproducibility.

### Animal infection model

To evaluate the pathogenic potential of *L. holotrichiae* isolates *in vivo*, a murine peritoneal infection model was established. Six-week-old female BALB/c mice (*n* = 18, specific-pathogen-free) were purchased from Beijing Vital River Laboratory Animal Technology Co., Ltd. and maintained under controlled conditions with *ad libitum* access to food and water. All procedures were approved by the Animal Ethics Committee of [Shihezi University], under protocol number [A2025-906]. Each bacterial isolate (LH23001, LH23002, and LH23003) was cultured overnight in BHI broth, washed, and resuspended in sterile PBS. Groups of six mice were intraperitoneal injected with 0.2 mL bacterial suspension containing 1 × 10^7^ CFU. A negative control group received sterile PBS alone. Mice were monitored daily for clinical signs including lethargy, weight loss, and local inflammation at the injection site. At 120 h post-inoculation, all mice were euthanized by CO_2_ asphyxiation. Gross pathological examination was performed, and visceral organ tissues were collected for histological analysis. The tissue was fixed in 10% neutral buffered formalin, embedded in paraffin, sliced 5 μm, and stained with hematoxylin and eosin (H&E). Each internal organ has different injuries. No clinical abnormalities or tissue lesions were observed in the PBS-injected controls.

### Whole-genome sequencing and annotation

Genomic DNA was extracted from overnight cultures of *Leucobacter holotrichiae* isolates LH23001, LH23002, and LH23003 using the TIANamp Bacteria DNA Kit (Tiangen Biotech, China), following the manufacturer’s instructions. DNA quality and concentration were assessed by agarose gel electrophoresis and a NanoDrop spectrophotometer (Thermo Fisher Scientific, United States). Whole-genome sequencing was performed on the Illumina NovaSeq 6,000 platform (Novogene, China), generating paired-end reads with an average insert size of 350 bp. Raw reads were quality-trimmed using Trimmomatic v0.39, and *de novo* genome assembly was conducted using SPAdes v3.15.3. Genome completeness and contamination were evaluated using CheckM v1.1.6. Gene prediction and functional annotation were performed using the NCBI Prokaryotic Genome Annotation Pipeline (PGAP) and Prokka v1.14.6 (Simpson et al., 2009; Xu et al., 2020; Besemer and Borodovsky, 2005; Lowe and Eddy, 1997; Lagesen et al., 2007). Protein-coding sequences were further annotated using multiple public databases, including COG (Clusters of Orthologous Groups), KEGG (Kyoto Encyclopedia of Genes and Genomes), VFDB (Virulence Factor Database), and CARD (Comprehensive Antibiotic Resistance Database). The circular genome map was visualized using CGView. The original sequencing genome sequences of strains LH23001, LH23002 and LH23003 have been deposited in the gene bank GenBank, and their accession numbers are, respectively, SAMN51023674, SAMN51023675, SAMN51023676. The complete genome assembly sequence is currently under review at NCBI, and the temporary accession number is SAMN52852392, SAMN52852393, SAMN52852394.

### Statistical analysis

All quantitative data are presented as mean ± standard deviation (SD) from at least three independent experiments. Statistical analyses were performed using GraphPad Prism version 9.5 (GraphPad Software, United States). Comparisons between multiple groups were conducted using one-way analysis of variance (ANOVA) followed by Tukey’s multiple comparisons test. For two-group comparisons, unpaired two-tailed Student’s *t*-tests were used. Differences were considered statistically significant at *p* < 0.05. For biofilm formation assays, OD values were compared across isolates using ANOVA. For antimicrobial susceptibility tests, zone diameters were analyzed descriptively and categorized based on CLSI guidelines, without inferential statistics. Histological severity scores from the animal infection model were semi-quantitatively evaluated but not statistically compared due to limited sample size. All statistical methods applied were pre-specified and consistent across replicates. Figures were generated using GraphPad Prism and Adobe Illustrator 2023 for final layout and annotation.

## Results

### Clinical and epidemiological findings

**Figure 1:**
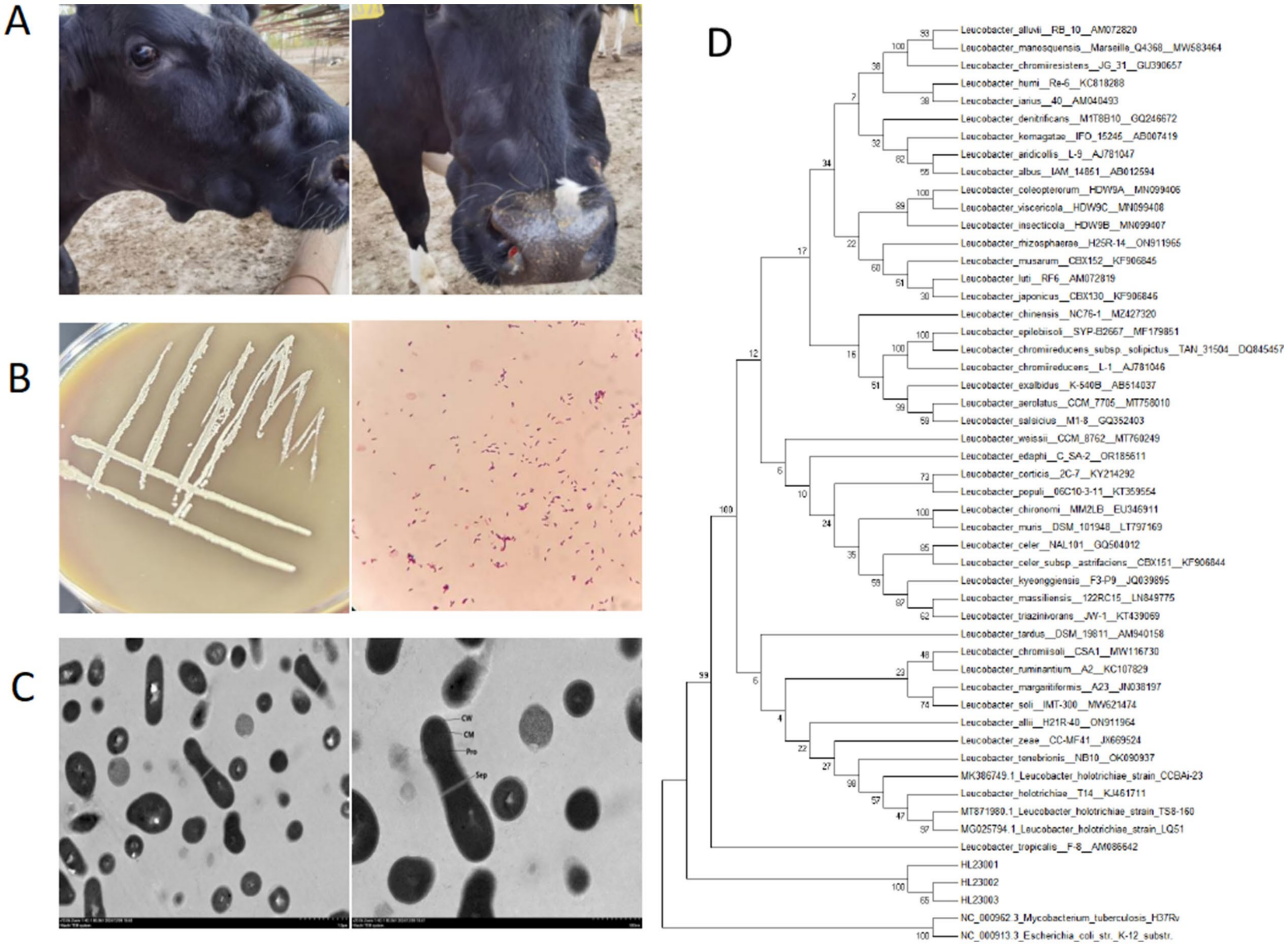
Isolation and identification of *Leucobacter holotrichiae*. Clinical presentation, colony morphology, and phylogenetic identification of *Leucobacter holotrichiae* **(A)** Submandibular actinomycotic abscess in dairy cattle. **(B)** Colony morphology in pure culture (blood agar medium) and gram staining morphology under light microscope (1000×). **(C)** Scanning electron microscope images (10,000×, 20,000×). **(D)** Neighbor-joining phylogenetic tree based on nearly full-length 16S rRNA sequences, showing the relationship of isolates LH23001, LH23002, and LH23003 to related *Leucobacter* species. Bootstrap values (50%) from 1,000 replicates are indicated at the nodes.

From April to July 2023, a total of 37 adult Holstein cows from three large farms in Changji, Xinjiang, China, presented with localized submandibular or mandibular swelling (Figure 1A). The clinical symptoms were confined to the affected area, with no obvious systemic signs such as fever or depression. Physical examination revealed that the swellings were solid, poorly defined, and had limited mobility. Among the affected cattle, 24 cases exhibited purulent exudate upon pressure or puncture. A total of 19 pus samples were collected using sterile syringes and submitted for laboratory analysis. Generally, the purulent material appeared as a viscous, yellow-white liquid, sometimes containing visible particles. The samples were inoculated onto blood agar and brain heart infusion (BHI) agar plates and cultured at 37 °C for 24 h. Bacterial colonies grew well under aerobic conditions and displayed mixed morphology. In the primary culture of the original samples, several distinct colony types were observed simultaneously on BHI agar, indicating a polymicrobial community within the abscesses. Preliminary 16S rRNA sequencing analysis of these different colonies identified other bacteria, including *Actinomyces lingnae*, *Staphylococcus aureus*, and *Corynebacterium pyogenes*. These microorganisms are common commensals or environmental flora on cattle skin or mucosa but are also known to have pathogenic potential. Among the mixed colonies, three isolates (designated LH23001, LH23002, and LH23003), obtained from different individual cows across the three farms, formed pale yellow, dry, and rough colonies that were distinct from others. Based on preliminary morphological characteristics and Gram staining, these isolates were tentatively classified as *Actinobacteria*. Conventional phenotypic and biochemical tests could not identify these microorganisms using commercial identification systems. 16S rRNA gene sequencing showed that the isolates shared over 99.8% identity with *L. holotrichiae* (Figure 1D). This species was previously known to colonize the intestinal tract of insects but has not been reported in mammalian infections. As this is the first isolation and identification of *L. holotrichiae* from a mammalian host, this study focuses on a detailed characterization of the strain to elucidate its biological features and potential pathogenic significance. This discovery also raises questions regarding its host range, environmental transmission, and pathogenic potential in vertebrates.

### Phenotypic and biochemical characteristics of isolates

**Figure 2:**
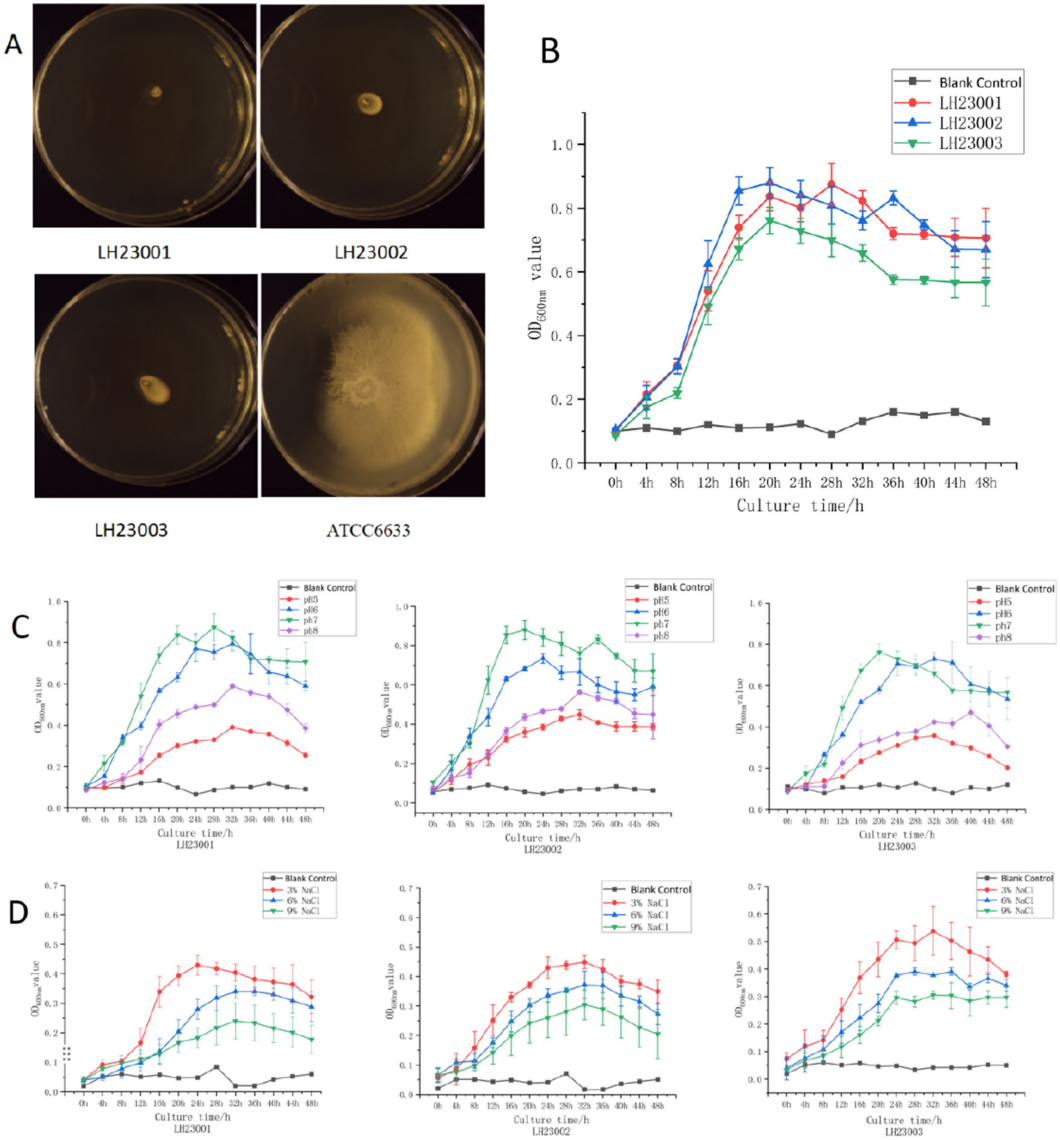
Phenotypic and physiological characteristics. Phenotypic and physiological characteristics of *L. holotrichiae* isolates. **(A)** Results of motility test of *L. holotrichiae* isolates. **(B)** Growth curve in BHI broth at 37 °C. **(C)** Growth curves in BHI broth at different pH concentrations. **(D)** Growth curves in BHI broth at different NaCl concentrations. Data represent the mean ± standard deviation of three independent experiments.

The three isolates, designated LH23001, LH23002, and LH23003, displayed consistent colony morphology. After 24 h of aerobic incubation at 37 °C on blood agar or BHI agar, colonies appeared dry, yellowish, wrinkled, and with rough surfaces, approximately 1–2 mm in diameter (Figure 1B) and were non-motile (Figure 2A). Transmission electron microscopy (Figure 1C) field of view showed that the bacterial morphology was mostly oval or short rod-shaped. No hemolytic activity was observed. Gram staining revealed that all isolates were Gram-positive short rods, arranged singly or in pairs, and non-spore-forming. Acid-fast staining was negative. All three isolates were catalase-positive and oxidase-negative. Biochemical identification was performed using the API Coryne test system (bioMérieux, France). Results showed that the strains were positive for pyrazinamidase, β-glucosidase, and glucose fermentation, and negative for urease, nitrate reduction, and gelatin hydrolysis. The API profile numbers did not match any known species in the system, yielding low discrimination scores. In terms of growth conditions, the isolates grew well in BHI broth supplemented with 5% fetal bovine serum (FBS) and tolerated NaCl concentrations up to 9% (Figure 2B). No growth was observed at 10% NaCl or under anaerobic conditions (Figure 2C). The isolates could grow in the environment of pH5-pH8, and the optimal growth pH was pH7 (Figure 2D). Based on their morphological and biochemical profiles, the isolates did not correspond to common bovine pathogens. Their colony features and API results suggested affiliation with high-GC Gram-positive actinobacteria, supporting the initial classification under the genus *Leucobacter*.

### Antibiotic susceptibility profiles

The antibiotic susceptibility profiles of *L. holotrichiae* strains LH23001, LH23002, and LH23003 were determined using the standard Kirby-Bauer disk diffusion method on Mueller-Hinton agar plates, incubated at 37 °C for 24 h under aerobic conditions. Results were interpreted based on Clinical and Laboratory Standards Institute (CLSI) guidelines where available: All three strains were Susceptible to commonly used β-lactam antibiotics, including penicillin (10 units), ampicillin (10 μg) and cefotaxime (30 μg), and their inhibition zone diameters were greater than 20 mm. It was also susceptible to tetracycline (30 μg), gentamicin (10 μg), enrofloxacin (5 μg), norfloxacin (10 μg) and chloramphenicol (30 μg). It showed moderate sensitivity to erythromycin (15 μg), and its inhibition zone diameter was between 16 and 20 mm. All strains were resistant to Benzylpenicillin (10 μg), Furazolidone (10 μg), Bacitracin (10 μg) and Clindamycin (2 μg), and the inhibition zone diameter was less than 13 mm. Antibiotic sensitivity is summarized in Table 1. These strains showed a consistent pattern, suggesting that they are inherently resistant mechanisms rather than acquired mutations.

**Table 1:** Antimicrobial susceptibility testing results for *Leucobacter holotrichiae* Strain.

Antibiotic	CLSI standard (S≥; I; R≤)	Inhibitory zone diameter/mm			Interpretation		
		Strain LH23001	Strain LH23002	Strain LH23003	Strain LH23001	Strain LH23002	Strain LH23003
Cefixime	≥19; 16–18; ≤15	27.6 ± 0.4	26.1 ± 0.3	27.3 ± 0.4	S	S	S
Amoxicillin	≥20; −; ≤19	28.4 ± 0.7	26.3 ± 0.5	24.3 ± 0.2	S	S	S
Neomycin	≥17; 14–16; ≤13	18.2 ± 0.3	16.0 ± 0.2	18.0 ± 0.3	S	I	S
Tetracycline	≥19; 16–18; ≤15	27.8 ± 0.6	26.0 ± 0.4	25.1 ± 0.2	S	S	S
Vancomycin	≥17; 15–16; ≤14	23.6 ± 0.5	22.8 ± 0.4	22.9 ± 0.3	S	S	S
Enrofloxacin	≥22; 19–21; ≤18	26.4 ± 0.2	26.0 ± 0.5	25.5 ± 0.7	S	S	S
Ofloxacin	≥16; 13–15; ≤12	22.2 ± 0.5	23.1 ± 0.3	21.3 ± 0.5	S	S	S
Roxithromycin	≥18; 14–17; ≤13	17.2 ± 0.5	16.8 ± 0.2	16.9 ± 0.4	I	I	I
Benzylpenicillin	≥32; 29–31; ≤28	6.0 ± 0.1	6.0 ± 0.1	6.0 ± 0.1	R	R	R
Furazolidone	≥16; 13–15; ≤12	12.1 ± 0.2	10.6 ± 0.2	11.8 ± 0.1	I	R	R
Bacitracin	≥13; 11–12; ≤10	6.0 ± 0.1	6.0 ± 0.1	6.0 ± 0.1	R	R	R
Clindamycin	≥19; 16–18; ≤15	6.0 ± 0.1	6.0 ± 0.1	6.0 ± 0.1	R	R	R

S, susceptible; I, intermediate; R, resistant.

### Biofilm-forming capacity

To assess their capacity to form biofilms, the three *L. holotrichiae* isolates (LH23001, LH23002, and LH23003) were cultured in 96-well polystyrene microtiter plates using BHI broth supplemented with 1% glucose. After 24 h of static incubation at 37 °C, adherent cells were stained with 0.1% crystal violet and quantified by measuring absorbance at 595 nm. All three isolates demonstrated the ability to form biofilms, with strain LH23001 exhibiting the highest OD < sub > 595</sub > value (1.21 ± 0.11), followed by LH23002 (0.96 ± 0.09) and LH23003 (0.63 ± 0.08). Based on standard classification criteria, LH23001 and LH23002 were categorized as moderate biofilm producers, while LH23003 exhibited weak biofilm-forming ability. One-way analysis of variance showed that there were significant statistical differences in biofilm formation ability among different isolates (*p* < 0.01), indicating that not all *Leucobacter* isolates have the same ability to form biofilm *in vitro*. From a biological point of view, this variation is very important because it means that some strains may have a stronger potential to colonize and persist on the host surface. The formation of biofilm is a key virulence factor of bacterial pathogenicity, which is related to the increased tolerance to antimicrobial therapy and the increased difficulty of immune system clearance. Specifically, the strong biofilm-forming ability of *L. holotrichiae* may help it establish and maintain chronic infection in primary abscess samples and mouse models.

### Pathogenicity in mice

**Figure 3:**
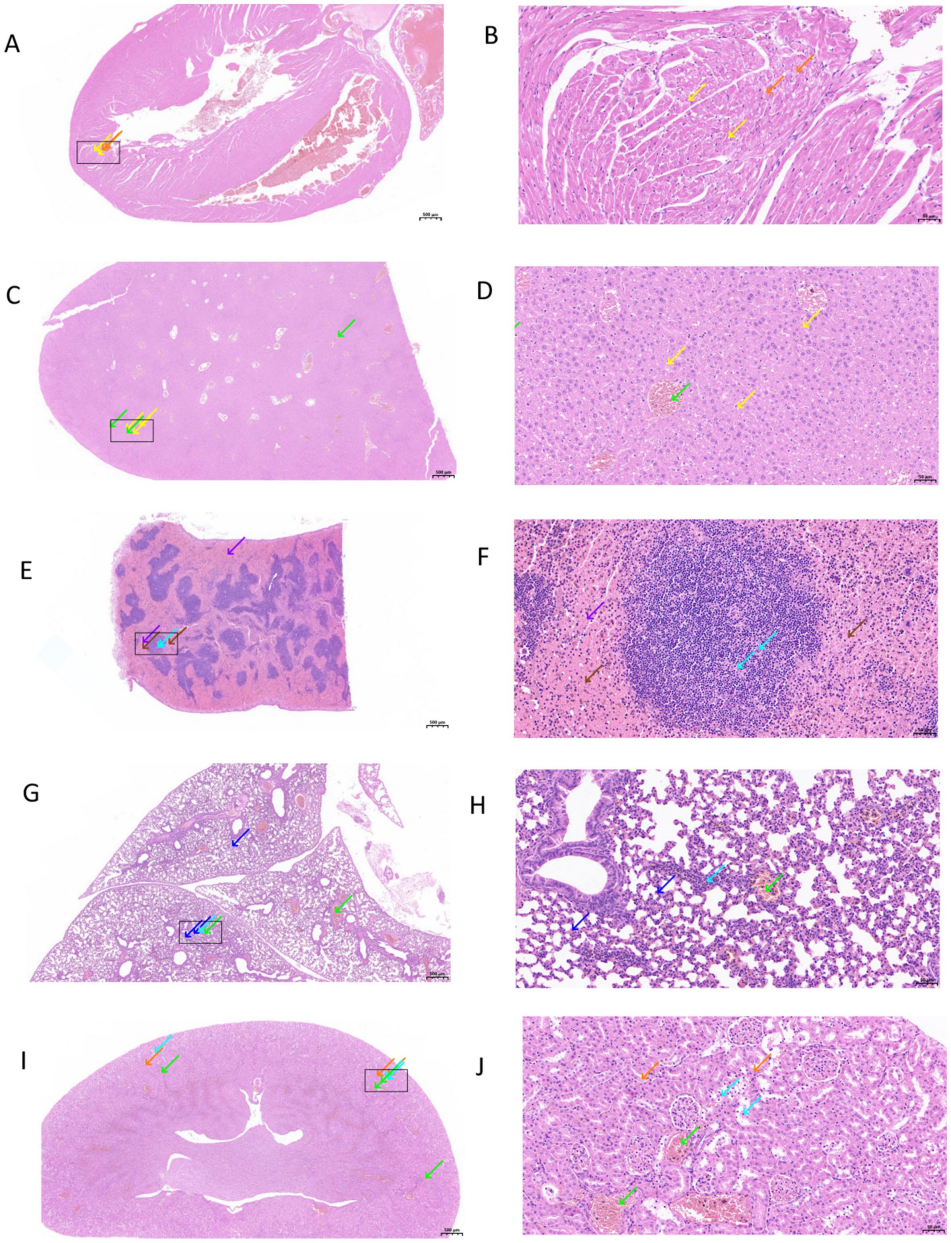
Pathogenicity in mice. Experimental infection of BALB/c mice with *L. holotrichiae*. **(A)** Histopathological changes in cardiac tissue (H&E staining, scale bar = 500 μm). The boxed area is the magnified region shown in **(B)**. **(B)** High-power field with a 50 μm scale bar in the same case. A small amount of myocardial cell vacuolar degeneration (yellow arrow), small vacuoles in the cytoplasm, and a small amount of myocardial cell edema (orange arrow) were observed. **(C)** Histopathological changes in liver tissue (H&E staining, scale bar = 500 μm). The boxed area is the magnified region shown in **(D)**. **(D)** High-power field with a 50 μm scale bar in the same case. Mild steatosis (yellow arrows) was observed in liver tissue cells, and small round vacuoles were seen in the cytoplasm. There was no obvious expansion or extrusion of hepatic sinus; more central vein and portal vein congestion (green arrow). **(E)** Histopathological changes in spleen tissue (H&E staining, scale bar = 500 μm). The boxed area is the magnified region shown in **(F)**. **(F)** High-power field with a 50 μm scale bar in the same case. It can be seen that a small amount of lymphocyte punctate necrosis (blue arrows) can be seen in the white pulp of the spleen tissue, and the nucleus is pyknosis and deep staining; a large range of red pulp congestion (purple arrow), red pulp visible amount of brown pigment deposition (brown arrow). **(G)** Histopathological changes in lung tissue (H&E staining, scale bar = 500 μm). The boxed area is the magnified region shown in **(H)**. **(H)** High-power field with a 50 μm scale bar in the same case. A large number of granulocyte infiltration (blue arrow) was seen in the alveolar wall of the lung tissue, and the alveolar wall was slightly thickened and the alveolar septum was widened. Interstitial visible more vascular lumen filled with granulocytes (cyan arrow), more vascular congestion (green arrow). **(I)** Histopathological changes in kidney tissue (H&E staining, scale bar = 500 μm). The boxed area is the magnified region shown in **(J)**. **(J)** High-power field with a 50 μm scale bar in the same case. It can be seen that there was more mild edema of renal tubular epithelial cells (orange arrows) in the cortex of renal tissue, loose and lightly stained cytoplasm, more necrosis and shedding of renal tubular epithelial cells (blue arrows), and pyknosis and deep staining of nucleus. Interstitial no obvious hyperplasia, more vascular congestion (green arrow).

To evaluate the virulence potential of *L. holotrichiae* isolates in a mammalian host, an intraperitoneal infection model was established using female BALB/c mice (6–8 weeks old, *n* = 5 per group). Each mouse was inoculated with 0.2 mL of bacterial suspension containing 1 × 10^7^ CFU of strains LH23001, LH23002, or LH23003, respectively. Control mice received sterile PBS. Throughout the 120 h observation period, infected mice exhibited transient signs of ruffled fur and decreased activity, particularly in the LH23001 group. No mortality was observed in any group. On 72 h post-infection, animals were euthanized for gross pathological and histological evaluation. Necropsy revealed visible fibrinous exudates and turbid ascites in the abdominal cavity of mice infected with LH23001 and LH23002, whereas the LH23003 group showed only mild peritoneal congestion. No lesions were observed in the control group. Histopathological examination (H&E staining) of liver, spleen, and kidney tissues showed inflammatory infiltration, hepatocellular vacuolation, and splenic red pulp expansion in LH23001- and LH23002-infected mice. Lesions were milder in the LH23003 group. Representative micrographs are shown in Figure 3.

### Genomic features of the isolates

**Figure 4:**
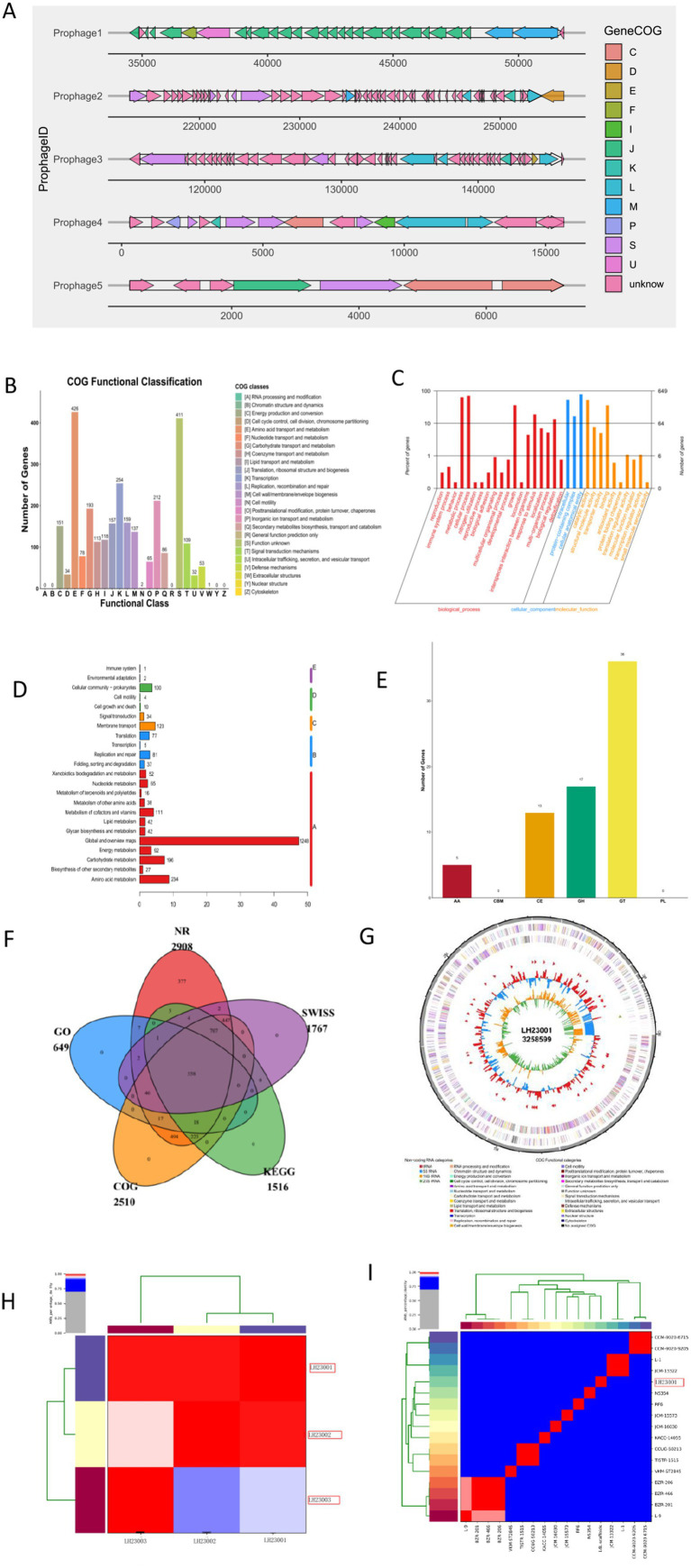
Genomic features and functional note. Genomic characteristics of *L. holotrichiae* isolates **(A)** Prophage prediction results. **(B)** Distribution of genes in different COG functional categories. **(C)** KEGG metabolic pathway annotation statistics. **(D)** GO database annotation diagram. **(E)** Annotated CAZy statistical chart. **(F)** Venn diagram of annotation results from five databases. **(G)** Circular genome map of strain LH23001 showing coding sequences (CDS), tRNAs, rRNAs, GC content, and GC skew. **(H)** The average nucleotide identity (ANI) among the three strains. **(I)** The average nucleotide identity (ANI) between the isolates and the reference species of the *Leucobacter*.

Whole-genome sequencing of *L. holotrichiae* strains LH23001, LH23002, and LH23003 was conducted on the Illumina NovaSeq platform. Quality assessment results of the genomic sequencing data are summarized in Table 2. The assembled genomes range in size from 3.63 to 3.68 megabases, with GC contents between 66.8 and 67.2%, and N50 values ranging from 85,000 to 96,000 base pairs. Gene prediction identified 3,200 to 3,280 coding sequences (CDS), including genes involved in oxidative stress resistance (e.g., catalase and superoxide dismutase), metal ion transport, and biofilm regulation. All three genomes contain genes encoding multidrug efflux pumps as well as various virulence-associated factors. Additionally, five active prophages were predicted. Comparative genomic analysis revealed an average nucleotide identity (ANI) greater than 99.6%, supporting a close genetic relationship among the isolates. BLAST analysis of the 16S rRNA gene and whole-genome sequences further confirmed their classification as *L. holotrichiae*, showing over 99.4% sequence identity with the reference strains TS8-160 (GenBank: MT871980.1) and LQ51 (GenBank: MG025794.1) (Figure 4).

**Table 2:** Sequencing data statistics table.

Sample ID	Raw data (Mb)	Clean data (Mb)	Clean data Q20 (%)	Clean data Q30 (%)	Clean data GC (%)
LH23001	1311.5	1,138	97.21	92.49	67.43
LH23002	1,286	1117.2	97.25	92.57	67.87
LH23003	1080.6	950	97.34	92.85	68.14

### Comparative genomic and functional note

The KEGG database annotation mapped 2,637 genes to 23 distinct pathways. The analysis demonstrated that *Leucobacter holotrichiae* possesses a pronounced genetic predisposition toward metabolic functions, with 2,163 genes (82.02% of KEGG-annotated genes) participating in diverse metabolic pathways. Amino acid metabolism constituted the most represented category (234 genes), followed by carbohydrate metabolism (196 genes) and metabolism of cofactors and vitamins (111 genes). This considerable gene repertoire underscores the organism’s substantial metabolic versatility.

The GO database annotation categorized 1,461 genes into functional classifications. Within the molecular function domain, catalytic activity and binding represented the most prevalent functional assignments. Cellular components were predominantly characterized by fundamental cellular architecture, while biological processes were chiefly represented by metabolic processes, cellular processes, and growth as the three most abundant annotations (Table 3).

**Table 3:** Statistical table of assembly results.

Sample ID	LH23001	LH23002	LH23003
Gene num	3,010	3,011	3,011
Gene total length	2,924,466	2,924,220	2,924,559
Gene average length	971.	971.	971.
Gene density genes per kb	0.923	0.923	0.924
GC content in gene region	69.3%	69.3%	69.3%
Gene/Geonme	89.7%	89.7%	89.7%
plus Gene num	1,346	1,351	1,332
minus Gene num	1,664	1,660	1,679
tRNA	48	48	48
5S rRNA	1	1	1
16S rRNA	1	1	1
23S rRNA	1	1	1

CAZy database analysis revealed 71 annotated genes, encompassing the following enzyme classes: auxiliary activities (5), carbohydrate esterases (13), glycoside hydrolases (17), and glycosyl transferases (36).

Comprehensive analysis using the Virulence Factor Database (VFDB) and the Comprehensive Antibiotic Resistance Database (CARD) detected 4 categories encompassing 8 resistance genes (Table 4). VFDB annotation identified 3 functional gene categories comprising 10 genes (Table 5), primarily implicated in bacterial metabolism, metal ion acquisition and homeostasis, immune evasion mechanisms, antioxidant defense and stress adaptation, alongside pathogenicity mechanisms associated with metabolic and structural functions.

**Table 4:** Drug resistance gene prediction results.

Primary mechanism	Sub-category	ARO name	Identity %	Coverage %
Antibiotic inactivation	Enzymatic modification	*novA*	*87.5*	94.6
	ABC transporter	*macB*	*81.0*	88.3
		*oleC*	*86.2*	92.8
	MFS transporter	*tetA*	*81.3*	95.2
Target protection	Ribosomal RNA methylation	*Erm*	*86.8*	90.5
Regulatory genes	Two-component system	*vanS*	*86.3*	94.5
		*vanR*	*81.3*	84.4
		*vanO*	*90.5*	94.0

**Table 5:** Virulence gene prediction results.

VF class	Virulence factors	Related genes	Identity %	Coverage %
Copper uptake	Copper exporter	*ctpV*	82.0	94.6
Iron uptake	ABC transporter	*irtA*	90.1	94.4
		*irtB*	86.0	97.4
	Exochelin (smegmatis)	*fxuB*	81.2	83.8
	ABC transporter (Corynebacterium)	*fagA*	80.3	94.0
Protease	Zn++ metalloprotease	*zmp1*	84.0	95.2
Regulation	PhoP/R	*phoP*	82.4	86.2
	RegX3	*regX3*	86.3	92.3
	Alpha-crystallin	*hspX*	80.2	94.7
	Catalase-peroxidase	*katG*	82.0	89.6

## Discussion

This study presents the first confirmed isolation of *L. holotrichiae* from a mammalian host, marking a notable expansion in the known ecological and host range of this species. Previously described exclusively from insect gastrointestinal environments, *L. holotrichiae* was generally considered a harmless environmental commensal with no reported pathogenicity in vertebrates. Our identification of three independent strains from actinomycotic abscesses in cattle challenges this assumption and highlights its potential role as an opportunistic pathogen. By combining clinical, microbiological, animal model, and genomic data, we provide compelling evidence that *L. holotrichiae* is not merely a transient contaminant, but a biologically competent organism capable of surviving—and possibly persisting—in vertebrate tissue. These findings contribute to the growing recognition that traditionally non-pathogenic actinobacteria may possess latent virulence traits and represent a previously underappreciated component of the emerging pathogen landscape in veterinary medicine.

The detection of *L. holotrichiae* in a vertebrate host is consistent with a broader trend in which environmental actinobacteria, once considered benign, are increasingly recognized as opportunistic pathogens under specific ecological or immunological conditions (Hyun et al., 2021; Zhu et al., 2016; Clark and Hodgkin, 2015). Similar shifts have been documented in related genera such as *Corynebacterium*, *Rhodococcus*, and *Gordonia*, many of which now occupy dual roles as both environmental saprophytes and conditional pathogens (Chen and Williamson, 2011). The expansion of this cognition is partly due to the wide application of high-throughput identification technology represented by next-generation sequencing, which enables us to more accurately identify the unusual or difficult-to-culture microorganisms in infected lesions. Therefore, the first appearance of L. holotrichiae in mammalian lesions may reflect its ecological expansion as a potential emerging pathogen, or it may be due to the improvement of detection ability to make it ‘visible’. Its ability to form moderate biofilms, tolerate oxidative and osmotic stress, and persist in murine tissues suggests that it is equipped with baseline traits necessary for survival in vertebrate niches (Zhu et al., 2022). Further genomic analysis revealed the genetic basis of host interactions such as adhesion-related genes, sorting enzyme homologs, and iron uptake systems, which are often considered to be part of the conditional pathogenic mechanism (Donlan and Costerton, 2002; Alekshun and Levy, 2007).

Genome analysis showed that the core metabolic framework of *L. holotrichiae* was particularly prominent, and a large number of genes were involved in the metabolic processes of amino acids, carbohydrates and cofactors. The enrichment of some key amino acid biosynthesis genes may contribute to its survival and reproduction in a nutrient-rich host environment (Tamakoshi et al., 1998; Zhang et al., 2012). In addition, there are diverse iron acquisition systems in the genes, indicating that they have the potential to reduce nutritional dependence and respond to the host’s ‘nutritional immunity’ (Alekshun and Levy, 2007; Rodriguez and Smith, 2006). In addition, the annotation of carbohydrate active enzyme (CAZyme) reveals its strong glycosylation ability, which is mainly reflected in the significant advantages of glycosyltransferases (GTs) (36/71, 50.7%). This enzyme system plays a key role in the biosynthesis of cell wall polysaccharides (such as peptidoglycan and lipopolysaccharide), indicating that its cell envelope integrity may be enhanced (Srivastava and Ghosh, 2025; Scaletti et al., 2023). These intrinsic metabolic plasticity, efficient nutrient acquisition mechanisms and enhanced physical barriers together constitute the genetic basis, which may together give *L. holotrichiae* a strong ability to maintain a competitive advantage in harsh environments such as bovine abscesses.

Although the pathogenic potential of *L. holotrichiae* remains to be fully elucidated, genomic analysis has identified homologs of multiple key virulence-associated genes, suggesting its capacity for host interaction and persistent colonization. It harbors a high-affinity iron acquisition system, including *irtA, irtB,* and *fxuB*, which may enable the bacterium to compete for essential iron in the host environment (Rodriguez and Smith, 2006; Ryndak et al., 2010). Additionally, genes such as *zmp1* and *ctpV* reveal its potential mechanisms of host damage and defense (Liang et al., 2021). Global regulatory systems, including *phoP* and *regX3*, may allow the bacterium to sense host-derived signals and adjust its gene expression accordingly (Zwir et al., 2005; Parish, 2003). Furthermore, the presence of *hspX* and *katG* indicates its ability to resist oxidative stress and other environmental pressures encountered during infection (Nono et al., 2025). The coexistence of these genetic traits, encompassing nutrient acquisition, immune regulation, stress response, and regulatory adaptation, suggests that *L. holotrichiae* is genetically equipped with tools facilitating its survival in mammalian hosts.

Genomic analysis further revealed that *L. holotrichiae* carries multiple antimicrobial resistance genes, indicating potential resistance to various drug classes. Bioinformatics predictions identified genes associated with resistance to fluoroquinolones (*novA*), macrolides (*Erm, macB, oleC*), tetracyclines (*tetA*), and glycopeptides (*vanS, vanR, vanO*). A significant discrepancy exists between the bacterium’s resistance genotype and phenotype, posing additional challenges for clinical drug use. On one hand, the clindamycin-resistant phenotype is fully consistent with the Erm resistance gene it carries, validating the predictive value of genotype for phenotype. On the other hand, despite harboring the *tetA* tetracycline efflux pump gene and glycopeptide resistance-related genes (*vanS, vanR, vanO*), the bacterium remains susceptible to tetracyclines and glycopeptides. This genotype–phenotype inconsistency may stem from complex factors at multiple biological and technical levels (Diarmaid and Andersson, 2017). At the genetic regulatory level, resistance genes may exhibit low basal expression levels or be in a state of transcriptional silencing, with their full activation often relying on induction by specific environmental signals (Fojo, 2007). At the gene function level, the gene sequences identified by bioinformatics predictions may contain nonsense or frameshift mutations in key functional domains, or the function of their expression products may depend on specific cofactors or genetic backgrounds, thereby preventing the formation of fully active resistance proteins. Therefore, the resistance genotype revealed by genomic data should be regarded as a potential resistance risk that may be activated under specific conditions (Ju et al., 2025).

The recovery of *L. holotrichiae* from actinomycotic abscesses in dairy cattle also prompts a re-evaluation of the microbial landscape underlying chronic suppurative infections. During the isolation process, we also isolated various other microorganisms, including *Actinomyces lingnae*, *Staphylococcus aureus*, and *Corynebacterium pyogenes*. Bovine actinomycosis has historically been attributed to *Actinomyces bovis* as a primary pathogen (Ishikawa et al., 2000; Bauer et al., 2006); however, increasing evidence suggests that polymicrobial interactions and secondary colonizers may significantly contribute to lesion development, persistence, and immune evasion (Szafrański et al., 2017; Routray et al., 2025). The ability of *L. holotrichiae* to persist in purulent lesions, resist multiple antibiotics, and form biofilms suggests that it may act as a co-pathogen or opportunistic invader that exacerbates the chronicity of infection (Penttinen et al., 2016; Kaushik et al., 2020). Its detection in multiple independent samples from distinct anatomical sites strengthens the case for its biological relevance rather than incidental presence. While its precise role in disease pathogenesis remains to be fully elucidated, these findings warrant broader microbial profiling of similar abscesses in cattle and other livestock species. Recognizing atypical taxa such as *L. holotrichiae* as contributors to chronic infections may improve diagnostic sensitivity and inform more effective therapeutic strategies in veterinary practice.

While this study provides the first insights into the genomic characteristics and pathogenic potential of *L. holotrichiae*, several limitations should be acknowledged. First, there are a variety of cultures in the sample, and the isolation of *L. holotrichiae* does not prove that the bacteria are the main pathogenic bacteria. Second, due to the limited number of available strains, genomic analysis is limited and comprehensive pan-genomic analysis cannot be performed. Our conclusions primarily rely on bioinformatic predictions and homology-based comparisons. Although we identified various gene homologs associated with virulence and antimicrobial resistance, it is important to note that some of these may represent conserved physiological components rather than specific pathogenicity determinants. Moreover, the functional significance of these genetic characteristics remains speculative. Additionally, although the mouse infection model used here provides preliminary evidence for *in vivo* colonization, it does not fully replicate the natural infection route, tissue specificity, or host-specific immune response of cattle. Employing stricter thresholds for sequence identity and coverage in future studies would enhance the specificity of such predictions. Second, the functional implications of these genomic features remain speculative, as they have not been functionally validated through molecular experiments such as targeted mutagenesis or transcriptomic analysis. Furthermore, while the murine infection model used here provided preliminary evidence of *in vivo* colonization, it does not fully recapitulate the natural routes of infection, tissue tropism, or host-specific immune responses in cattle. Therefore, the assessment of its pathogenic potential and ecological role remains provisional. Future investigations should expand epidemiological surveillance, incorporate a greater diversity of isolates to decipher genomic plasticity, and employ integrated approaches—including functional genetics, *in vivo* gene expression analysis, and CRISPR-based functional assays—to elucidate the mechanistic basis of its pathogenicity and define the roles of key genes in the adaptation and virulence of *L. holotrichiae.*

## Conclusion

This study is the first to isolate and identify the species *Leucobacter holotrichiae* from mixed cultures of bovine actinomycotic abscesses. This finding significantly expands the known host range of this species and suggests its potential role as a novel opportunistic pathogen. Through an integrated approach combining microbiological phenotypic characterization, a murine infection model, and whole-genome sequencing, we demonstrated that this bacterium is capable of colonizing and persisting in a mammalian host. Key attributes supporting this capability include robust biofilm formation, broad stress tolerance, and a genetic repertoire harboring various features potentially involved in host interaction, such as adhesion, iron acquisition, and cell envelope fortification systems. It is crucial to emphasize that this bacterium was isolated from a polymicrobial abscess context. Therefore, the precise pathogenic role of *L. holotrichiae* in bovine actinomycosis—whether it acts as a primary pathogen, a co-pathogen, or a secondary colonizer—remains to be fully elucidated. In conclusion, our research broadens the understanding of the ecological diversity within the genus *Leucobacter* and underscores the importance of ongoing surveillance of environmental actinobacteria. Under specific conditions, such microorganisms may cross species barriers and emerge as potential sources of infection in veterinary medicine and even public health. Future investigations employing functional genomics, epidemiological surveys, and polymicrobial infection models are warranted to definitively establish the pathogenicity of *L. holotrichiae* and decipher its true transmission cycle in nature.

## Data Availability

The datasets presented in this study can be found in online repositories. The names of the repository/repositories and accession number(s) can be found in the article/supplementary material.
